# Supplementary material for: Content-rich biological network constructed by mining PubMed abstracts
Source: BMC Bioinformatics. 2004 Oct 8;5:147. doi: 10.1186/1471-2105-5-147 (PMC528731; doi:10.1186/1471-2105-5-147)
Supplement: Additional File 2 — The original results of the above study (non-essential files are deleted to keep the file size under the limit set by BMC bioinformatics). [file 1471-2105-5-147-S2.bz2 › chilibotAdditionalFile2/dip50/305ID10428074E1302/html/APP_APOE.html]

 


 **APP** and **APOE** 
  
Found 392 abstracts in PubMed, retrieved 50.  
 

 What does Google say? 
 PDF only 
| .edu only 

---

**Interactive relationship** (e.g. stimulation, inhibition, etc)

**Neutral relationship**- Niemann Pick type C disease accelerated neurofibrillary tangle formation and amyloid beta  [ **APP** ]  deposition associated with apolipoprotein E  [ **APOE** ]  epsilon 4 homozygosity.  Ref: 12205649 Ann Neurol, 2002
- We analyzed murine  **apoE**  deficient transgenic mice expressing in their brains human  **APP**  and Abeta together with apoE3 or apoE4.  Ref: 12486146 J Neurosci, 2002
- **ApoE**  has been shown essential for amyloid beta  [ **APP** ]  peptide fibrillogenesis and deposition, a defining pathological feature of this disease.  Ref: 12468034 Brain Res, 2002
- RESULTS Compared with those in the same aged mice, in the 5 month old SAM, levels of gene expression of MR, tau, PS 2 and  **APP**  were significantly higher, that of apo E  [ **APOE** ]  lower, levels of gene expression PS 1 and bcl 2 were unobviously changed.  Ref: 12572383 Zhongguo Zhong Xi Yi Jie He Za Zhi, 2002
- The incidence was significantly higher in atherosclerotic  **apoE**  deficient  **apoE**  mice, in which 8 100% of 8 mice developed AAA  [ **APP** ] .  Ref: 12600880 Circ Res, 2003
- Phosphatidylcholine phospholipase D activity, cell membrane cholesterol, and apolipoprotein E  [ **APOE** ]  levels were associated with effects of fresh amyloid beta  [ **APP** ]  peptide 1 42on cholesterol distribution but not with effects of aged amyloid beta  [ **APP** ]  peptide 1 42arguing against a common mechanism.  Ref: 12584199 J Biol Chem< MedlineTA>J Biol Chem, 2003
- Here we show that, in contrast to  **APP**  Tg mice expressing  **apoE** ,  **APP**  Tg mice deficient in  **apoE**  develop anti Abeta immunoreactive multifocal aggregates, which contain the beta cleaved C terminal fragments of  **APP** .  Ref: 12419536 Brain Res, 2002
- Mutations in  **APP**  and the presenilin genes account for less than 1% of the prevalence of the disease in the general population compared to 10 17% for the  **APOE**  variation.  Ref: 12629906 Tijdschr Gerontol Geriatr, 2003
- Possession of the  **APOE**  varepsilon 4 allele has also been shown to influence neuropathological findings in patients who die from traumatic brain injury, including the accumulation of amyloid beta  [ **APP** ]  protein.  Ref: 12782758 Mol Pathol< MedlineTA>Mol Pathol, 2003
- Tg mice deficient in  **apoE**  also display altered levels of mature full length  **APP** , increased amounts of beta CTFs, as well as elevated levels of Abeta and Abeta in an age and region dependent manner when compared to Tg mice expressing  **apoE** .  Ref: 12419536 Brain Res, 2002

**Non-interactive relationship** (e.g. studied together, co-existance, homology, etc.)

- Amyloid beta  [ **APP** ]  peptide, Abeta precursor protein, apolipoprotein E  [ **APOE** ] , and elevated cholesterol levels have been linked to Alzheimer s disease pathology.  Ref: 12206998 FASEB J, 2002
- The reason for unchanged levels of CSF amyloid beta  [ **APP** ]  and CSF  **apoE**  after ischemic stroke remains unclear.  Ref: 12214084 J Alzheimers Dis< MedlineTA>J Alzheimers Dis, 2002
- Ang II induced AAA  [ **APP** ]  in 9 90% of 10 hyperlipidemic mice deficient in  **apoE**   **apoE**  uPA mice but in only 2 22% of 9 mice deficient in both  **apoE**  and uPA  **apoE**  uPA mice P< ; 0.05.  Ref: 12600880 Circ Res, 2003
- while in the 12 month old SAM, gene expression of MR and tau were higher, bcl 2 was lower and PS 1, PS 2,  **apoE**  and  **APP**  were also unobviously changed.  Ref: 12572383 Zhongguo Zhong Xi Yi Jie He Za Zhi, 2002
- METHODS Changes of learning and memory related gene expression, including mineralocorticoid receptor, presenile protein 1 and 2, tau,  **APP** ,  **apoE**  and bcl 2 in hippocampus of SAM were determined by reverse transcription polymerase chain reaction.  Ref: 12572383 Zhongguo Zhong Xi Yi Jie He Za Zhi, 2002
- Apolipoprotein E  [ **APOE** ]  enhances uptake of soluble but not aggregated amyloid beta  [ **APP** ]  protein into synaptic terminals.  Ref: 12614344 J Neurochem, 2003
- Taken together, these data support a role for  **apoE**  in  **APP**  processing in vivo.  Ref: 12419536 Brain Res, 2002
- Most of these studies have examined only one or two polymorphisms in a defined population, which provides a lack of a general view of the Alzheimer s disease genetic component.OBJECTIVETo overcome this limitation, nine polymorphisms in seven different candidate genes A2M, ACT,  **APOE** ,  **APP** , BH, HSP70 2, and IL1 A were genotyped.METHODSThe sample comprised 112 Alzheimer s disease patients and 89 controls from Spain.  Ref: 12782964 Psychiatr Genet, 2003
- In contrast, CSF amyloid beta  [ **APP** ]  and CSF  **apoE**  showed no significant changes during the period.  Ref: 12214084 J Alzheimers Dis< MedlineTA>J Alzheimers Dis, 2003
- We have examined the effect of apolipoprotein E  [ **APOE** ]  on the internalization of exogenous amyloid beta  [ **APP** ]  1 40 Abeta40 into a rat brain crude synaptosomal preparation.  Ref: 12614344 J Neurochem, 2003
- We have also found that while proteins that accumulate in Alzheimer disease such as tau, amyloid beta  [ **APP** ] , and apolipoprotein E  [ **APOE** ]  possess metal binding sites, metal associated cellular redox activity is more dependent on metal nucleic acid binding.  Ref: 12572666 Biometals, 2003
- We have recently reported a critical role for apolipoprotein E  [ **APOE** ]  in the process of amyloid deposition and neuritic plaque formation in  **APP**  transgenic mice, an animal model of Alzheimer s disease.  Ref: 12419536 Brain Res, 2002
- The  **APOE** \*4P mutation was observed in heterozygous state in four subjects, two AD 1  [ **APP** ] .71% and two controls 1.65%.  Ref: 12815750 Am J Med Genet, 2003
- The present study was undertaken to test the hypothesis that estrogen can attenuate Ang II induced AAA  [ **APP** ]  in apolipoprotein E  [ **APOE** ]  deficient mice via its anti inflammatory mechanism.  Ref: 12855485 Arterioscler Thromb Vasc Biol< MedlineTA>Arterioscler Thromb Vasc Biol, 2003
- Mann Neuropathology and Applied Neurobiology 2 5 44 Tau load is associated with apolipoprotein E  [ **APOE** ]  genotype and the amount of amyloid beta  [ **APP** ]  protein, Abeta40, in sporadic and familial Alzheimer s disease The total amount of hyperphosphorylated tau protein, present as neurofibrillary tangles, neuropil threads or plaque neurites, was quantified in the frontal cortex of 109 cases of sporadic Alzheimer s disease and 35 cases of familial AD due to missense mutations in the presenilin 1, presenilin 2 and amyloid precursor protein genes.  Ref: 12581338 Neuropathol Appl Neurobiol< MedlineTA>Neuropathol Appl Neurobiol, 2003
- High cholesterol levels increase  **APP**  and  **apoE**  expression in human NT2 neuron progenitor cells.  Ref: 12206998 FASEB J, 2002
- Potential cerebrospinal fluid markers for Alzheimer s disease include tau protein, the 42 amino acid form of amyloid beta  [ **APP** ]  and apolipoprotein E  [ **APOE** ] .  Ref: 12214084 J Alzheimers Dis< MedlineTA>J Alzheimers Dis, 2002
- A well defined ultrastructural characteristic within Alzheimer dense plaques is the presence of helical fibrils that are believed to consist of polymerized amyloid beta  [ **APP** ] , together with other associated proteins such as the serum amyloid P protein, apolipoprotein E  [ **APOE** ]  isoform epsilon 4, alpha1 anti chymotrypsin, catalase, glycoproteins, proteoglycans, cholesterol and other lipids.  Ref: 12475558 Micron, 2002
- Cerebral levels of total  **APP**  were not altered while cerebral  **apoE**  levels were increased in the mice fed the Western type diet, versus the chow fed mice.  Ref: 12474021 J Nutr Health Aging, 2002
- Interestingly, beta protein precursor, amyloid beta  [ **APP** ] , presenilins, and apolipoprotein E  [ **APOE** ]  have all been linked to reactive oxygen species production or apoptosis, a process intimately associated with oxidative stress.  Ref: 12214011 J Alzheimers Dis< MedlineTA>J Alzheimers Dis, 2002
- We have previously demonstrated that urokinase type plasminogen activator uPA is highly expressed in the aneurysmal segment of the abdominal aorta AAA  [ **APP** ]  in apolipoprotein E  [ **APOE** ]  deficient  **apoE**  mice treated with angiotensin II Ang II.  Ref: 12600880 Circ Res, 2003
